# Differentiating Between Multiple Myeloma and Metastasis Subtypes of Lumbar Vertebra Lesions Using Machine Learning–Based Radiomics

**DOI:** 10.3389/fonc.2021.601699

**Published:** 2021-02-24

**Authors:** Xing Xiong, Jia Wang, Su Hu, Yao Dai, Yu Zhang, Chunhong Hu

**Affiliations:** ^1^ Department of Radiology, The First Affiliated Hospital of Soochow University, Suzhou, China; ^2^ Institute of Medical Imaging, Soochow University, Suzhou, China; ^3^ State Key Laboratory of Radiation Medicine and Protection, Soochow University, Suzhou, China

**Keywords:** metastasis, multiple myeloma, machine learning, magnetic resonance imaging, vertebra

## Abstract

**Objective:**

To determine whether machine learning based on conventional magnetic resonance imaging (MRI) sequences have the potential for the differential diagnosis of multiple myeloma (MM), and different tumor metastasis lesions of the lumbar vertebra.

**Methods:**

We retrospectively enrolled 107 patients newly diagnosed with MM and different metastasis of the lumbar vertebra. In total 60 MM lesions and 118 metastasis lesions were selected for training classifiers (70%) and subsequent validation (30%). Following segmentation, 282 texture features were extracted from both T1WI and T2WI images. Following regression analysis using the least absolute shrinkage and selection operator (LASSO) algorithm, the following machine learning models were selected: Support‐Vector Machine (SVM), K-Nearest Neighbor (KNN), Random Forest (RF), Artificial Neural Networks (ANN), and Naïve Bayes (NB) using 10-fold cross validation, and the performances were evaluated using a confusion matrix. Matthews correlation coefficient (MCC), sensitivity, specificity, and accuracy of the models were also calculated.

**Results:**

To differentiate MM and metastasis, 13 features in the T1WI images and 9 features in the T2WI images were obtained. Among the 10 classifiers, the ANN classifier from the T2WI images achieved the best performance (MCC = 0.605) with accuracy, sensitivity, and specificity of 0.815, 0.879, and 0.790, respectively, in the validation cohort. To differentiate MM and metastasis subtypes, eight features in the T1WI images and seven features in the T2WI images were obtained. Among the 10 classifiers, the ANN classifier from the T2WI images achieved the best performance (MCC = 0.560, 0.412, 0.449), respectively, with accuracy = 0.648; sensitivity 0.714, 0.821, 0.897 and specificity 0.775, 0.600, 0.640 for the MM, lung, and other metastases, respectively, in the validation cohort.

**Conclusions:**

Machine learning–based classifiers showed a satisfactory performance in differentiating MM lesions from those of tumor metastasis. While their value for distinguishing myeloma from different metastasis subtypes was moderate.

## Introduction

Bone metastasis and multiple myeloma (MM) are two different diseases, although both frequently involve bone marrow evaluation during clinical workup (1), which may result in bone pain and fractures for patients (2). Metastasis is the most common outcome of tumors and is often displayed as an osteolytic or sclerosing lesion on bone tissue (3). To identify metastasis, ^18^F-Fluorodeoxyglucose (^18^F-FDG) Positron Emission Tomography (PET) and Computed Tomography (CT) (^18^F-FDG PET/CT) play irreplaceable roles in detecting primary cancer and evaluating metastasis, but are accompanied by high radiation exposure and expensive costs for patients. For example, metastases from lung cancer are the most prevalent type of metastases (4). If these lesions were accurately predicted by conventional magnetic resonance imaging (MRI), it would narrow the examination range to using chest CT, which is easily accessible and much cheaper. The identification of cheaper imaging examinations to detect primary cancer will thus provide a beneficial cost-effective approach for the management of patients. Recently, the morbidity of MM has increased (5, 6). Although MM can be adequately monitored by quantifying paraproteins (M-protein) in the serum and urine, some myelomas are non-secretory or hypo-secretory and are therefore difficult to manage after the primary diagnosis (7). Thus, precise identification of vertebra lesions using medical images could be beneficial for follow-up examinations and treatment strategies. In particular, for patients who do not have a known primary cancer, a correct diagnosis would provide important information for choosing the most appropriate clinical workup. Chemotherapy and radiation therapy are the two main options for the treatment of myeloma patients (8). With regard to metastatic cancer, further follow-up for detecting the primary cancer may be needed before choosing the optimal treatment strategy, which may include surgery, radiation, and/or chemotherapy. While MRI can provide detailed morphological information about lesions and is the most sensitive imaging modality for tumor infiltration in bone marrow, MM and metastasis appear similar and are often indistinguishable (9), particularly for multiple vertebra focal osteolytic lesions (10). Previous studies have reported that vascular parameters measured by dynamic-contrast-enhanced (DCE) MRI can help identify primary spinal cancers (11, 12) and metastatic cancers of different primary tumors (13, 14).

Machine learning is an emerging area of “radiomics” that extracts, analyzes, and interprets quantitative imaging features and has been applied in many fields (15–17). Machine learning allows for objective evaluation of lesions and organ heterogeneity beyond a subjective visual interpretation and may provide valuable information about the tissue microenvironment (18). Machine learning algorithms are categorized into supervised (using labeled data) and unsupervised (using unlabeled examples) learning and are able to process a large number of radiomic variables to characterize tumor phenotypes. The goal of supervised learning is to learn from a certain portion of a trained data set with known labels and to predict the classification for unknown patterns from datasets using algorithms such as Support Vector Machine (SVM), Random Forest (RF), and Artificial Neural Network (ANN).

To the best of our knowledge, there have been no studies to date focusing on the differential diagnosis of MM and metastasis subtypes on lumbar vertebra based on conventional MRI sequences. This study aimed to determine whether machine learning–based classifiers could be helpful to differentiate MM lesions on lumbar vertebra from metastatic lesions and their respective subtypes.

## Material and Methods

### Patients

**Figure 1:**
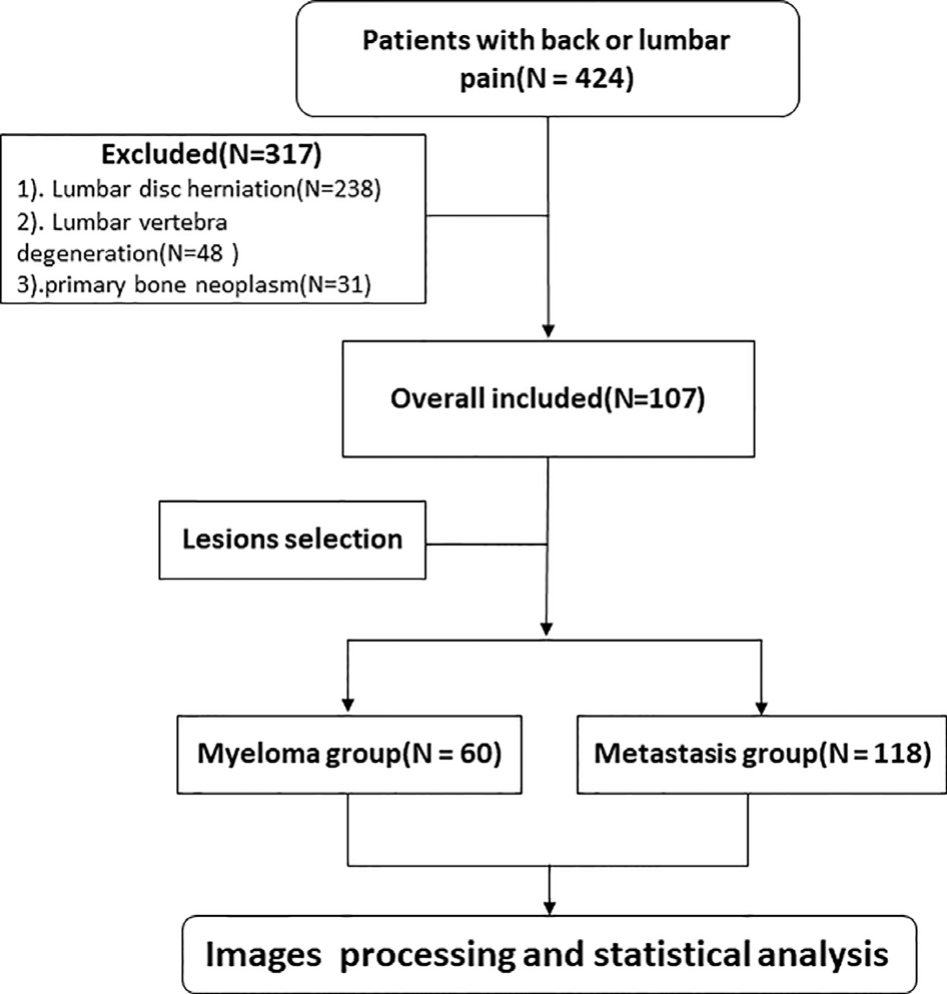
The flowchart of inclusion and exclusion criteria.

This study was approved by the Ethics Committee of our institution and the need for informed consent was waived. We retrospectively collected clinical and MRI information of patients experiencing back or lumbar pain from January 2018 to May 2020. Inclusion criteria: (1) patients diagnosed with MM according to the International Myeloma Working Group Diagnostic Criteria (19) or metastatic tumors on lumbar vertebra confirmed by core needle or excisional biopsy; (2) patients with no MRI examination contradiction; (3) patients with intact and high quality MRI images before treatment, including sagittal T1WI and sagittal and transverse T2WI sequences; (4) at least one lesion having a diameter >1 cm; and (5) availability of complete clinical information. Exclusion criterion: (1) patients presenting solely lumbar disc herniation; (2) patients presenting solely spinal degenerative changes; and (3) patients with primary bone neoplasm. All patients in the study had no prior history of malignant tumor diagnosis, and all metastasis patients had been subjected to pathological analyses for primary cancer. The eligible patients were randomly divided into the training and validation cohorts at a ratio of 7 to 3. The flowchart shows the analysis pathway for this study (**Figure 1**).

### MRI Examination

All patients underwent MRI examinations using a 3.0T MRI scanner (Magnetic Verio, Siemens Healthcare, Erlangen, Germany) equipped with a Total imaging matrix system. The protocol included the following parameters: sagittal T1W turbo spin echo (TSE) (repetition time/echo time, 1,700 ms/8.6 ms; section thickness, 4 mm; gap, 0.8 mm; turbo factor, 8; FOV, 448 mm × 448 mm), sagittal T2W TSE fs (repetition time/echo time, 3,000 ms/91 ms; section thickness, 4 mm; gap, 0.8 mm; turbo factor, 15; FOV, 448 mm × 448 mm), as well as transverse T2W TSE (repetition time/echo time, 4,040 ms/100 ms; section thickness, 4 mm; gap, 0.8 mm; turbo factor, 25; FOV, 348 mm × 384 mm). The scanning region ranged from T11 to S1.

### Lesion Segmentation

**Figure 2:**
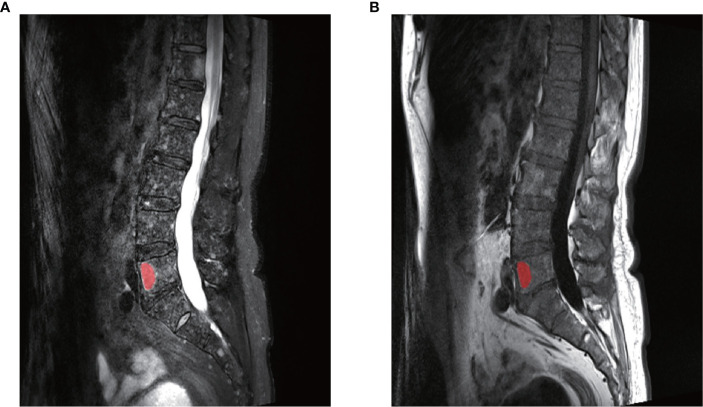
An example of the manual segmentation in one lesion with myeloma. **(A)** The segmented area was within the red contour on the largest cross-sectional area on sagittal T2WI. **(B)** The segmented area was copied to the same slice of T1WI images. The two slices were from the same patient at the same axis.

All images were collected from the institution’s Picture Archiving and Communication System (PACS) in the form of DICOM with accordant window width and window location. The region of interest (ROI) was created manually from T2WI using MaZda (version 4.6.0, Institute of Electronics, Technical University of Lodz). Only lesions with hypointensity on the T1W TSE images and corresponding intermediate to high signal intensity on T2W TSE fs images were selected for analysis. Since there may be multiple lesions on each patients’ lumbar vertebra, only the lesions whose diameters were >1 cm were selected to avoid the partial volume effect. Meanwhile, if the number of lesions on the vertebra meeting the requirements were more than 3, then the largest of the 3 lesions was chosen for the analysis. The detailed procedures were as follows: ROI were manually defined along the largest cross-sectional area on the sagittal T2W TSE fs in MaZda carefully avoiding the edge of the vertebra, Schmorl nodule, vessels, and vertebral hemangiomas. Classical vertebral hemangiomas are usually displayed as high-signal intensities both on T1W TSE and T2W TSE fs images. Next, the ROIs of the T2W TSE fs images were copied to the same location of the T1W TSE sequence (**Figure 2**).

### Texture Feature Extraction

Before feature extraction, gray-scale normalization was performed between μ ± 3σ (where μ, the mean value of the gray levels within the ROI; σ, the standard deviation) to reduce brightness and contrast variations and minimize the influence of inter-scanner as well as field strength differences, in order to improve the robustness and repeatability of texture features, as in previous studies (20, 21). Each lesion was jointly selected by two radiologists (one having 3 years’ experience and was reassessed by another senior radiologist with 10 years’ experience). They were both blinded to the clinical results.

As many as 282 variables were generated within each ROI, which were derived from 5 different statistical image descriptors: histogram features, gradient features, gray-level co-occurrence matrix (GLCM), gray-level run-length matrix (GRLM), and an autoregressive model (AR). GLCM and GRLM features were calculated at 6 bits per pixel, gradient features were calculated at 4 bits per pixel, the first-order histogram and the AR features were calculated at 8 bits per pixel. A detailed description of these textural features can be found at the official MaZda website (https://www.Eletel.p.Lodz.Pl/mAzda/).

### Feature Selection

All features were first normalized by subtracting the mean value and divided by the standard deviation. Then, to evaluate the reproducibility and stability of the features, another radiologist with 7 years’ experience independently segmented the ROIs in 30 randomly selected patients. All radiologists were blinded to the clinical information. Intraclass correlation coefficient (ICC) values were calculated for each texture feature. Only the features with ICC value ≥ 0.80 were termed as excellent reproducibility and were selected for further analysis. Then the least absolute shrinkage and selection operator (LASSO) regression method was performed for each classifier based on binomial deviance minimization criteria in the train cohort. A 10-fold cross-validation method was adopted to avoid potential bias.

### Classification and Validation

Five supervised machine‐learning algorithms were implemented in this study: SVM, RF, Naïve Bayes (NB), K-Nearest Neighbor (KNN), and ANN. A combination of two sequences, a total of 10 machine‐learning classifiers were constructed in the train cohort and tested in the validation cohort. For each model, 10‐fold cross‐validation was used to verify the classification accuracy in the train cohort, and the Matthews correlation coefficient (MCC, Eq. 1) of the generated confusion matrix was applied to quantify the differentiation performance in function of its robustness in the imbalanced data, as previously reported (22, 23). Accuracy, specificity, and sensitivity were also calculated.

$$MCC=[(TP\times TN)-(FP\times FN)]/{\left[(TP+FP)(TP+FN)(TN+FP)(TN+FN)\right]}^{1/2}$$

Eq.1. The equation of MCC; MCC, Matthews correlation coefficient; TP, true positive; TN, true negative; FP, false positive, FN, false negative.

### Statistical Analysis

Statistical tests were performed using R statistical software (version 3.3.3, https://www.r-project.org). Student’s t‐test or Mann-Whitney U test was applied for the continuous variables, and the χ^2^ test was applied for the categorical variables between the two cohorts as appropriate. A value of two-tailed P < 0.05 was regarded as statistically significant in this study.

## Results

### Patients

Overall, 107 patients were enrolled in the study, which included 60 patients with metastases (37 males, 23 females; age, 61.5 ± 8.6 years old) and 47 patients with MM (29 males, 18 females; age, 59.5 ± 10.9 years old). According to the International Staging System classification, MM were 8 in stage I, 25 in stage II, and 14 in stage III. Distribution of primary tumor included: lung cancer (n = 30), stomach cancer (n = 2), hepatocellular carcinoma (n = 2), renal cell carcinoma (n = 1), nasopharyngeal cancer (n = 13), rectal cancer (n = 1), and breast cancer (n = 11). Among them, 60 MM lesions and 118 metastasis lesions were selected for the training and validation of classifiers. There was no statistically significant difference in age or sex distribution between the training (n = 75) and validation (n = 32) cohorts (P = 0.910, 0.268, respectively).

### Analysis of Feature Reproducibility

In T1WI images, 194 out of 282 features showed excellent reproducibility (ICC ≥ 0.80). In T2WI images, 232 out of 282 features showed excellent reproducibility (ICC ≥ 0.80). Therefore, these features were accepted for further analysis.

### Diagnostic Performance Between Myeloma and Metastasis

**Figure 3:**
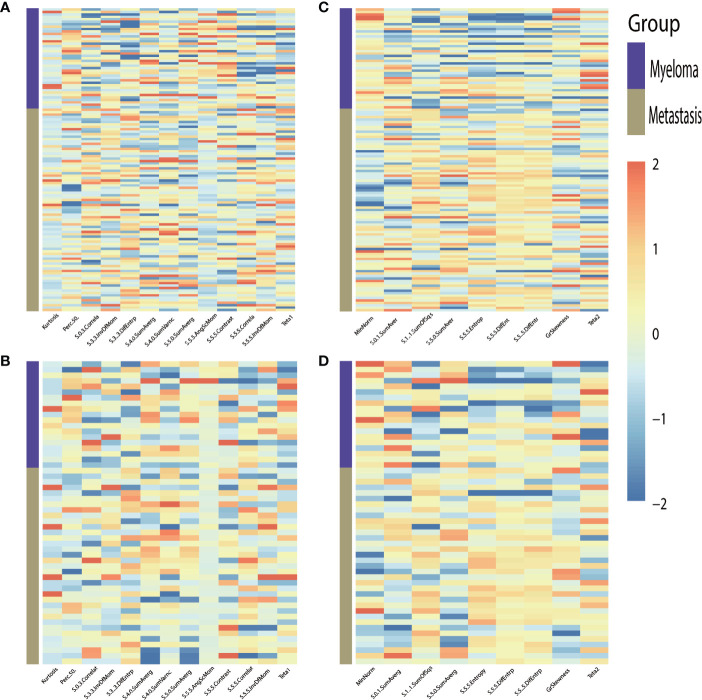
Heat-maps of the selected features from T1WI **(A, B)** and T2WI **(C, D)** for train **(A, C)** and validation **(B, D)** cohort show distribution and differences of normalized (z-score) feature values by presenting each lesion’s individual value.

For the classification of myeloma and metastasis, 13 features in T1WI images and 9 features in T2WI images were generated using the LASSO algorithm. The selected features and their values are presented in **Table 1** and **Figure 3**.

**Table 1:** Selected features for classification.

Sequence	Classification between myeloma and metastasis		Classification between myeloma and metastasis subtypes	
**T1WI**	Kurtosis	50th percentile	50th percentile	S(5.5)DifEntropy
	S(0.3)Correlation	S(3.3)InvDfMom	S(0.4)InvDfMom	S(4.4)InvDfMom
	S(3.3)DifEntropy	S(4.0)SumAverg	S(0.5)AngScMom	S(5.5)AngScMom
	S(4.0)SumVariance	S(5.0)SumAverg	S(5.5)Contrast	S(5.5)Entropy
	S(5.5)AngScMom	S(5.5)Contrast		
	S(5.5)Correlation	S(5.5)InvDfMom		
	Teta1			
**T2WI**	MinNorm	S(0.1)SumAverg	MinNorm	S(4.4)DifEntropy
	S(1.1)SumOfSqs	S(5.0)SumAverg	S(1.1)SumOfSqs	S(5.5)Entropy
	S(5.5)Entropy	S(5.5)DifEntropy	S(3.3)SumVariance	S(5.5)DifEntropy
	S(5.5)DifEntropy	GrSkewness	S(4.4)SumVariance	
	Teta2			

**Figure 4:**
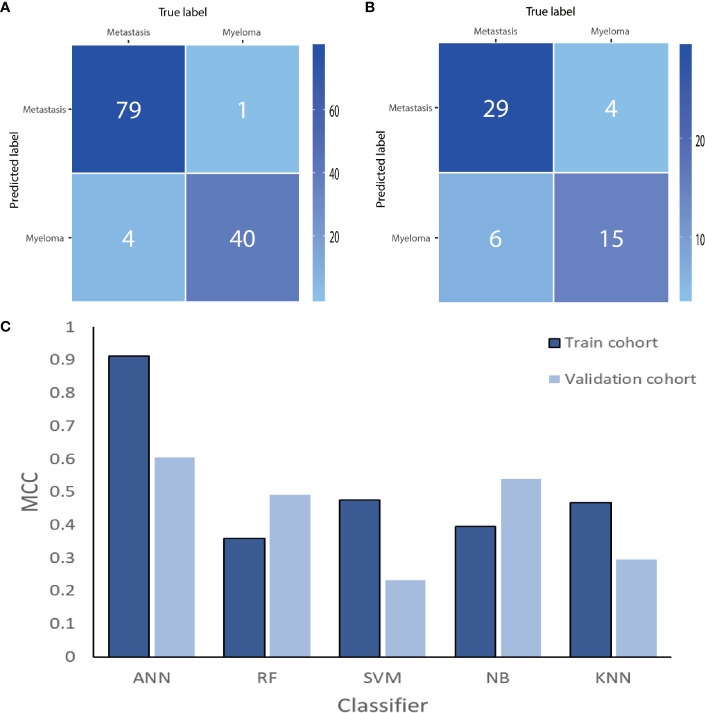
The ANN-based confusion matrix of train **(A)** and validation **(B)** cohort. Histogram **(C)** shows the performance of classifiers from T2WI images for discriminating myeloma and metastasis in train and validation cohort. ANN, artificial neural network; RF, random forest; SVM, support vector machine; NB, Naive Bayesian; KNN, K-nearest neighbor; MCC, Matthews correlation coefficient.

After cross-validation training, the ANN-based classifiers from T1WI and T2WI images achieved optimal performance with an MCC and accuracy value of 0.965, 0.912 and 0.960, 0.984, respectively. While in the validation cohort, the ANN-based classifier from T2WI images outperformed the other classifiers with an MCC and accuracy value of 0.605 and 0.815, respectively (**Table 2**, **Figure 4**). **Figure 4** shows the ANN-based confusion matrix generated for the training and validation cohorts and the performance of five classifiers from T2WI images.

**Table 2:** Classification results of machine learning–based classifiers in differentiating myeloma and metastasis.

Sequence	Classifier	Train cohort				Validation cohort			
		ACC	SEN	SPE	MCC	ACC	SEN	SPE	MCC
**T1WI**	**ANN**	0.984	1.000	0.954	0.965	0.685	0.764	0.550	0.318
	**RF**	0.742	0.880	0.462	0.329	0.704	0.886	0.368	0.301
	**SVM**	0.774	0.843	0.634	0.484	0.704	0.829	0.474	0.381
	**NB**	0.790	0.916	0.537	0.502	0.648	0.829	0.316	0.166
	**KNN**	0.734	0.268	0.964	0.345	0.704	1.000	0.158	0.329
**T2WI**	**ANN**	0.960	0.988	0.909	0.912	0.815	0.879	0.790	0.605
	**RF**	0.726	0.831	0.512	0.359	0.778	0.914	0.526	0.492
	**SVM**	0.774	0.988	0.342	0.475	0.685	0.943	0.211	0.233
	**NB**	0.750	0.904	0.439	0.396	0.796	0.943	0.526	0.539
	**KNN**	0.766	0.342	0.976	0.468	0.704	0.914	0.316	0.295

ANN, artificial neural network; RF, random forest; SVM, support vector machine; NB, Naive Bayesian; KNN, K-nearest neighbor; ACC, accuracy; SEN, sensitivity; SPE, specificity; MCC, Matthews correlation coefficient.

### Diagnostic Performance for Myeloma and Metastasis Subtypes

**Figure 5:**
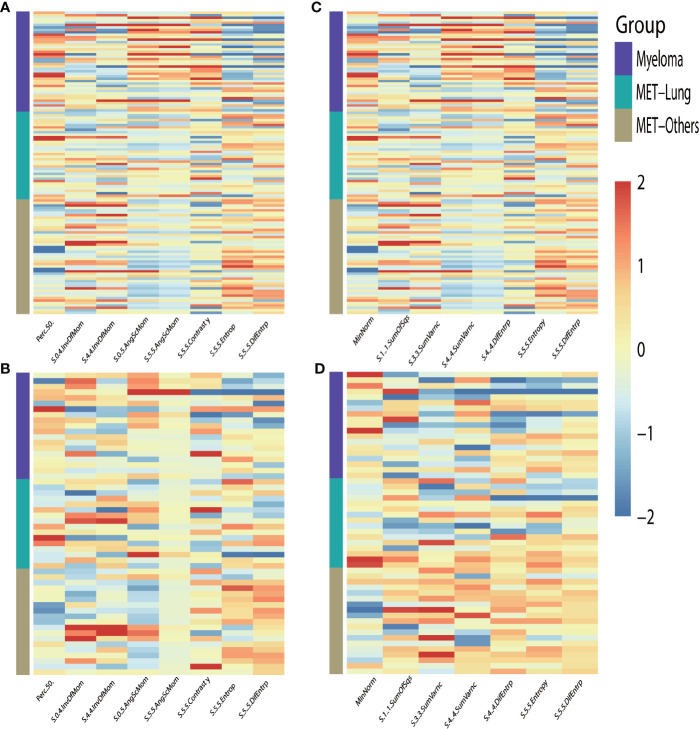
Heat-maps of the selected features from T1WI **(A, B)** and T2WI **(C, D)** for train **(A, C)** and validation **(B, D)** cohort show distribution and differences of normalized (z-score) feature values by presenting each lesion’s individual value; MET, metastasis.

To differentially classify myeloma from metastasis from lung cancer (Met-Lung) and metastasis from other tumors (Met-Others), 8 features in TIWI images and 7 features in T2WI images were identified using the LASSO method. The selected features and their values are presented in **Table 1** and in **Figure 5**.

**Figure 6:**
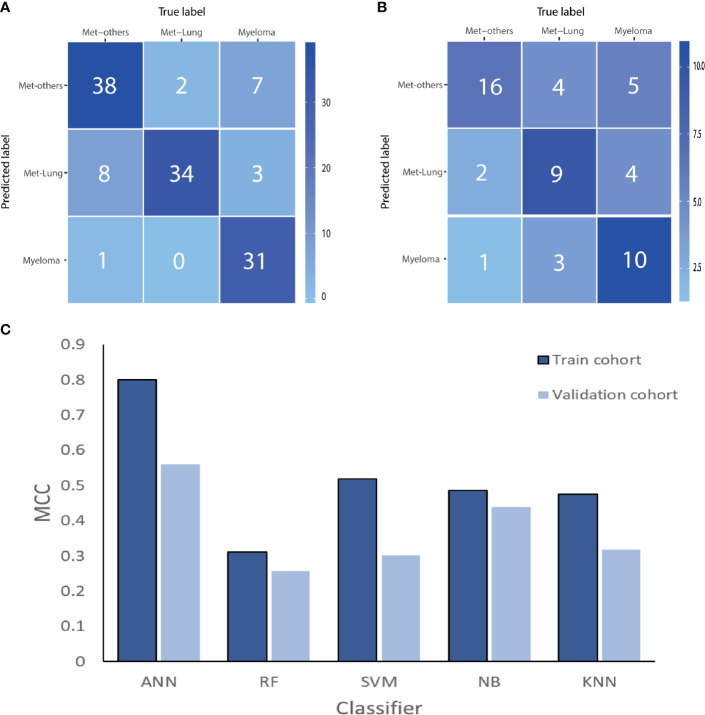
The ANN-based confusion matrix of train **(A)** and validation **(B)** cohort. Histogram **(C)** shows the performance of classifiers for discriminating myeloma from MET-Lung and MET-Others in train and validation cohort. MET, metastasis; ANN, artificial neural network; RF, random forest; SVM, support vector machine; NB, Naive Bayesian; KNN, K-nearest neighbor; MCC, Matthews correlation coefficient.

After cross-validation training, the ANN-based classifiers from T1WI and T2WI images achieved the optimal performance with MCC = 0.818, 0.704, 0.631, accuracy = 0.750 and MCC = 0.800, 0.774, 0.692, accuracy = 0.831, respectively (**Table 3**). While in the validation cohort, the ANN-based classifier from T2WI images outperformed the other classifiers with MCC = 0.560, 0.412, 0.449 and accuracy = 0.648, respectively (**Table 3**, **Figure 6**). To differentiate myeloma lesions from metastasis, the ANN-based classifier from T2WI images achieved a better performance in comparison with differentiating Met-Lung or Met-Others lesions from others tumor lesions in both the training and validation cohorts. **Figure 6** shows the ANN-based confusion matrix obtained for the training and validation cohorts and the performance of five classifiers from T2WI images.

**Table 3:** Classification results of machine learning–based classifiers in differentiating myeloma and metastasis subtypes.

Sequence	Classifier	Train cohort				Validation cohort			
		ACC	SEN	SPE	MCC	ACC	SEN	SPE	MCC
**T1WI**	**ANN**	0.750	0.970	0.901	0.818	0.519	0.588	0.767	0.336
			0.704	0.825	0.704		0.758	0.381	0.148
			0.656	0.917	0.631		0.763	0.625	0.324
	**RF**	0.492	0.537	0.735	0.267	0.551	0.588	0.865	0.470
			0.857	0.250	0.138		0.694	0.389	0.083
			0.642	0.674	0.302		0.771	0.684	0.446
	**SVM**	0.621	0.707	0.747	0.436	0.556	0.467	0.769	0.231
			0.389	0.875	0.299		0.765	0.450	0.223
			0.723	0.805	0.525		0.800	0.737	0.526
	**NB**	0.565	0.439	0.916	0.400	0.537	0.625	0.895	0.542
			0.417	0.727	0.141		0.771	0.316	0.095
			0.787	0.701	0.474		0.629	0.684	0.299
	**KNN**	0.581	0.537	0.892	0.465	0.518	0.474	0.857	0.360
			0.306	0.852	0.181		0.250	0.816	0.075
			0.830	0.610	0.430		0.790	0.600	0.373
**T2WI**	**ANN**	0.831	0.969	0.891	0.800	0.648	0.714	0.775	0.560
			0.756	0.975	0.774		0.821	0.600	0.412
			0.809	0.883	0.692		0.897	0.640	0.449
	**RF**	0.476	0.585	0.735	0.311	0.481	0.579	0.686	0.257
			0.194	0.807	0.002		0.188	0.816	0.004
			0.595	0.662	0.253		0.632	0.714	0.336
	**SVM**	0.637	0.707	0.819	0.518	0.556	0.526	0.771	0.302
			0.444	0.898	0.387		0.313	0.816	0.141
			0.723	0.727	0.509		0.790	0.743	0.512
	**NB**	0.589	0.561	0.892	0.487	0.574	0.526	0.829	0.440
			0.278	0.875	0.185		0.375	0.895	0.317
			0.8511	0.597	0.440		0.790	0.629	0.400
	**KNN**	0.597	0.659	0.819	0.475	0.519	0.580	0.743	0.318
			0.222	0.932	0.221		0.125	0.895	0.033
			0.830	0.623	0.442		0.790	0.620	0.400

ANN, artificial neural network; RF, random forest; SVM, support vector machine; NB, Naive Bayesian; KNN, K-nearest neighbor; ACC, accuracy; SEN, sensitivity; SPE, specificity; MCC, Matthews correlation coefficient.

## Discussion

In this study, machine learning–based MRI classifiers were constructed to establish a noninvasive classification of MM and metastasis subtypes of lumbar vertebra. The ANN-based classifier from T2WI images achieved satisfactory performances for differentiating myeloma from metastasis and moderate performance for classifying metastasis subtypes. To our knowledge, this is the first study to establish machine learning–based classifiers using conventional MRI sequences to distinguish MM and metastasis subtypes. The analysis revealed the value of machine learning–based classifiers from T2WI images in discriminating malignant tumors of lumbar vertebra.

Prior radiological study reported that compare with MM, bone metastases more commonly affect the vertebral pedicles rather than vertebral bodies, rarely involve mandible and distal axial skeleton (24). Uygar et al. (25) have compared the CT features of MM and osteolytic metastatic bone lesions. The result confirmed that the presence of high density, lesional homogeneity, perilesional sclerosis, and marginal features could be used to distinguish metastatic from MM lesions. Lee et al. (26) found that the salt and pepper infiltration pattern, the presence of more than five lesions within one vertebra, and the involvement of more than three consecutive vertebrae on MRI images were useful findings for differentiation between MM and metastasis involving the spine, but there were no significant differences in signal intensities or enhancement patterns. They concluded that it is difficult to distinguish between the two conditions in most cases. In additional, these conventional radiological features were assessed visually, so they depend on readers’ subjective evaluation and are not always typical.

Considering the difficulty of classification based on conventional radiological features, in recent years, advanced MRI technologies have been gradually applied to the differential diagnosis of focal vertebral lesions. Park et al. (1) held that the value of ADCav, ADCmin, and ADCvol of MM were significantly lower than those of metastasis. This study suggested that the addition of axial DWI to standard MR imaging can be helpful to diagnose MM from metastasis at 3T. In Lang et al.’s study (27), the characteristic DCE parameters between the myeloma and metastatic cancer groups were compared, and the findings showed that the myeloma group had a significantly higher Ktrans and Kep compared to the metastatic cancer group. Based on these findings, Lang et al. explored how to differentiate metastatic lesions in the spine that originated from primary lung cancer from other cancers using radiomics and deep learning based on DCE-MRI (28). However, advanced imaging is not included in all medical conditions and places high demands on acquisition and analysis methods. Thus, the ability to classify vertebra tumors based on conventional MRI sequences would be beneficial for clinical work-up.

Recently, radiomics has been proposed as an approach to overcome the limitations of visual assessment and has become a promising tool in modern radiology. By extracting and analyzing high throughput of image features, radiomics can provide important information about tissue physiology. A method that combines radiomics and machine learning has produced a non-invasive classification and prediction model able to distinguish histological subtypes of lesions (29, 30), distant metastasis of tumors (31), and therapeutic response or prognosis (32). In the current study, feature selection was performed by the LASSO method, which had proven to be efficient and effective for feature dimensionality reduction (33).

The results of feature selection showed that the most contributory features to the classification between subtypes derive from GLCM. This feature set is calculated by the number of gray-level combinations of images, distances, and angles (34), which reflect the local heterogeneity changes inside the lesion, as previous studies have reported (35–37). Compared with metastases, spinal myelomas have high cellular density with little interstitial space in histological level (27). Hence, myelomas should have lower heterogeneity in theory than metastases, which could explain the different gray-level distribution between spinal myelomas and metastases. For instance, the entropy reflects the regularity of texture and uniformity of grey-level distribution (38). Consistent with higher heterogeneity, the entropy of metastases from T2WI images was higher than that of myelomas in our study.

Classifiers were trained using various machine-learning algorithms including ANN, SVM, k-NN, NB, and RF in our study. Prior to validation, each classifier underwent further internal cross-validation to assess the classification accuracy. The best classifier was obtained using the ANN algorithm in T2WI images, regardless of differentiating myeloma from metastasis or subtypes. It indicates that compared with T1WI, T2WI contains more valuable texture features for identifying metastasis and myeloma. This may be because the echo time of T2WI is longer than T1WI, which increases the contrast between tissues, thus providing more information for identifying tissue heterogeneity (39). Universally applied in medical practice (40, 41), the ANN algorithm has proven its robust ability against a variety of input features and random noise (42). There is no universal optimal learning algorithm for all fields. Nevertheless, the classifiers constructed in the current study showed ANN’s capability of distinguishing myeloma from metastasis and subtypes of lumbar vertebrae with moderate to excellent performance.

Our study has several limitations. First, this was a retrospective study so the selection bias cannot be fully avoided; however, the current major radiomics or machine learning studies are retrospective in nature. Secondly, the classifiers built into this study were validated with internal data but were not tested with an external dataset due to the relatively small number of patients. Thirdly, considering the limitations of lesion size, only two-dimensional features were analyzed. Three-dimensional features of tumors may be more comprehensive and representative, but would be too time-consuming for routine clinical workup and is sensitive to the partial volume effect. Fourthly, our study achieved only moderate efficiency for differentiating MM and metastasis subtypes. Though in our opinion, compared to contrast-enhanced T1-weighted sequenced and functional sequences, such as DWI and DCE, conventional sequences provide limited information for tissue heterogeneity and the tumor microenvironment. However, conventional sequences are included for almost all standard MRI protocols, so the developed radiomics method is generalizable and feasible for application in clinical practice. Considering the errors involved in subjective evaluation, the diagnostic performance of MM compared to metastasis with conventional MRI sequences has not been calculated. More advanced sequences and conventional MRI features may be selected for further prospective studies. Moreover, demographic characteristic and laboratory examination results were excluded in current study, model combined clinical information and radiomics may improve the efficiency of the test. Finally, in clinical practice, not every lesion is pathologically confirmed. Nevertheless, we believe that this bias may be effectively avoided by using strict inclusion and exclusion criteria.

## Conclusion

Our findings demonstrate the satisfactory performance of machine learning methods based on conventional MRI sequence data to differentiate newly diagnosed myeloma lesions from metastatic lesions localizing on the lumbar vertebra. While the performance in distinguishing myeloma and metastasis subtypes is moderate, machine learning classifiers could potentially be valuable tools for optimizing precision medicine applied to lumbar vertebra tumors, and protecting patients from unnecessary exposure to radiation or examinations.

## Data Availability Statement

The raw data supporting the conclusions of this article will be made available by the authors, without undue reservation.

## Ethics Statement

The studies involving human participants were reviewed and approved by the Ethics Committee of the First Affiliated Hospital of Soochow University. Written informed consent for participation was not required for this study in accordance with the national legislation and the institutional requirements.

## Author Contributions

CH and YZ: guarantor of the article. JW and XX: conception, design, collection, and assembly of data. SH and YD: data analysis and interpretation. All authors contributed to the article and approved the submitted version.

## Funding

This study has received funding by the Project of State Key Laboratory of Radiation Medicine and Protection, Soochow University (No. GZK1201916), National Key Research and Development Program of China (No. 2017YFC0114300) and National Natural Science Foundation of China (No. 81771885).

## Conflict of Interest

The authors declare that the research was conducted in the absence of any commercial or financial relationships that could be construed as a potential conflict of interest.
